# Injectable Phosphorescence-based Oxygen Biosensors Identify Post Ischemic Reactive Hyperoxia

**DOI:** 10.1038/s41598-017-08490-0

**Published:** 2017-08-15

**Authors:** Jennifer S. Chien, Mahmoud Mohammed, Hysem Eldik, Mohamed M. Ibrahim, Jeremy Martinez, Scott P. Nichols, Natalie Wisniewski, Bruce Klitzman

**Affiliations:** 1 Kenan Plastic Surgery Research Labs and Biomedical Engineering, Duke University Medical Center, Durham, NC 27710 USA; 2 Profusa, Inc., 345 Allerton Ave, South San Francisco, CA 94080 USA

## Abstract

Novel injectable biosensors were used to measure interstitial oxygenation before, during, and after transient ischemia. It is well known that reactive hyperemia occurs following a period of ischemia. However, increased blood flow does not necessarily mean increased oxygen tension in the tissue. Therefore, the purpose of this study was to test the hypothesis that tissue reactive hyperoxia occurs following release of hind-limb tourniquet occlusions. Rats were injected with bilateral hind-limb biosensors and were simultaneously subjected to a unilateral femoral vessel ligation. After approximately one and three months, the rats underwent a series of oxygenation challenges, including transient hind-limb tourniquet occlusion. Along with the biosensors, near infrared spectroscopy was used to measure percent oxyhemoglobin in capillaries and laser Doppler flowmetry was used to measure blood flow. Post-occlusion reactive hyperemia was observed. It was accompanied by tissue reactive hyperoxia, affirming that the post-occlusion oxygen supply must have exceeded the expected increased oxygen consumption. The measurement of the physiologic phenomenon of reactive hyperoxia could prove clinically beneficial for both diagnosis and optimizing therapy.

## Introduction

Disorders characterized by perfusion compromise such as stroke, myocardial infarction, and peripheral vascular disease remain the most frequent causes of debilitation and death. Both the magnitude and the duration of ischemia determine the severity and reversibility of cell injury and death^1^. Therefore, in order to predict and minimize the extent of ischemic injury, understanding the time course and intensity of the body’s response to a hypoxic insult remains a crucial area of investigation.

One way to characterize such a response is by examining the behavior of blood flow upon release of an arterial occlusion. The classical physiologic response immediately following hypoxic challenge is a transient increase in blood flow termed reactive hyperemia. During the period of occlusion, low perfusion pressure reduces vascular wall tension and leads to a local myogenic response causing vasodilation. In addition, it tips the balance of vasoactive metabolites, such as adenosine, in favor of arteriolar vasodilation that decreases local microvascular resistance. Upon restoration of perfusion pressure, the reduced microvascular resistance leads to elevated blood flow. The wall tension is restored, the vasodilatory metabolites are washed out and the tissue is re-oxygenated, causing the vascular tone and blood flow to gradually return to baseline^2^.

One method of monitoring blood flow in localized tissues is by laser Doppler flowmetry (LDF). This method is based on the frequency shift of backscattered laser light, which occurs as a result of the Doppler shift produced by the interaction of the light with moving cells (mostly erythrocytes) in the tissue^3–5^. In several *in vitro* systems, the LDF signal has been shown to be proportional to the product of the number of erythrocytes moving within the illuminated volume and the average velocity of the cells^4, 5^. In this study, we utilized LDF as a measure of characterizing blood flow in response to experimental modulations^6^. Although blood flow is one of the contributors to the supply of oxygen, it alone cannot adequately represent the oxygen content within the tissue due to concurrent variations in cellular oxygen consumption.

Upon dissociating from the red blood cell hemoglobin and diffusing into the extra-vascular space, the partial pressure of O_2_ within the interstitial tissue is sensitive to the dynamic balance between cellular oxygen supply and demand^7–9^. During the ischemic period, an oxygen debt accumulates within the affected tissue. Consequently, increased oxygen consumption is required to restore baseline homeostasis. The post-occlusion elevated blood flow and resulting increased oxygen supply could be counteracted by the tissue’s elevated oxygen consumption and extraction. To truly understand whether the supply meets, exceeds or falls short of the tissue’s demand after an ischemic insult, a direct measurement of the local tissue oxygenation is required.

To monitor oxygenation within tissues, one current standard technique is near infrared (NIR) spectroscopy. By spectrally analyzing changes in the relative concentrations of oxyhemoglobin and deoxyhemoglobin within tissues while minimizing the signal contribution from pulsatile arterial vessels, NIR spectroscopy estimates the percent oxygen saturation of hemoglobin in non-pulsatile vessels (veins and capillaries) within a large volume of tissue at various depths^10^. Thus, the non-invasive transcutaneous NIR interface and real-time continuous measurements provide important means to indirectly assess hemodynamics from tissue oxyhemoglobin. Some current clinical applications for NIR spectroscopy include the measurement of regional cerebral oxygenation, transcutaneous surgical flap viability, and skeletal muscle monitoring as an indicator of systemic stress^11–15^. However, one limitation of integrating data over the volume of tissue throughout a depth is the inclusion of tissue unrelated to the targeted area of interest (e.g., extra-cerebral tissue)^12^. In addition, pain and hypothermia-mediated vasoconstriction, as well as vasoactive agents such as phenylephrine or norepinephrine can also significantly influence extracranial blood contribution and subsequently alter NIR results^16^. Other factors, such as differences in hemoglobin distribution between arterial, venous and capillary systems, false readings from myoglobin within the overlying fat and muscle, or interfering changes between the sensor and light source, such as occurs following tissue edema, can all influence NIR spectroscopy readings that may not necessarily reflect the true state of tissue oxygenation^17, 18^.

**Figure 1:**
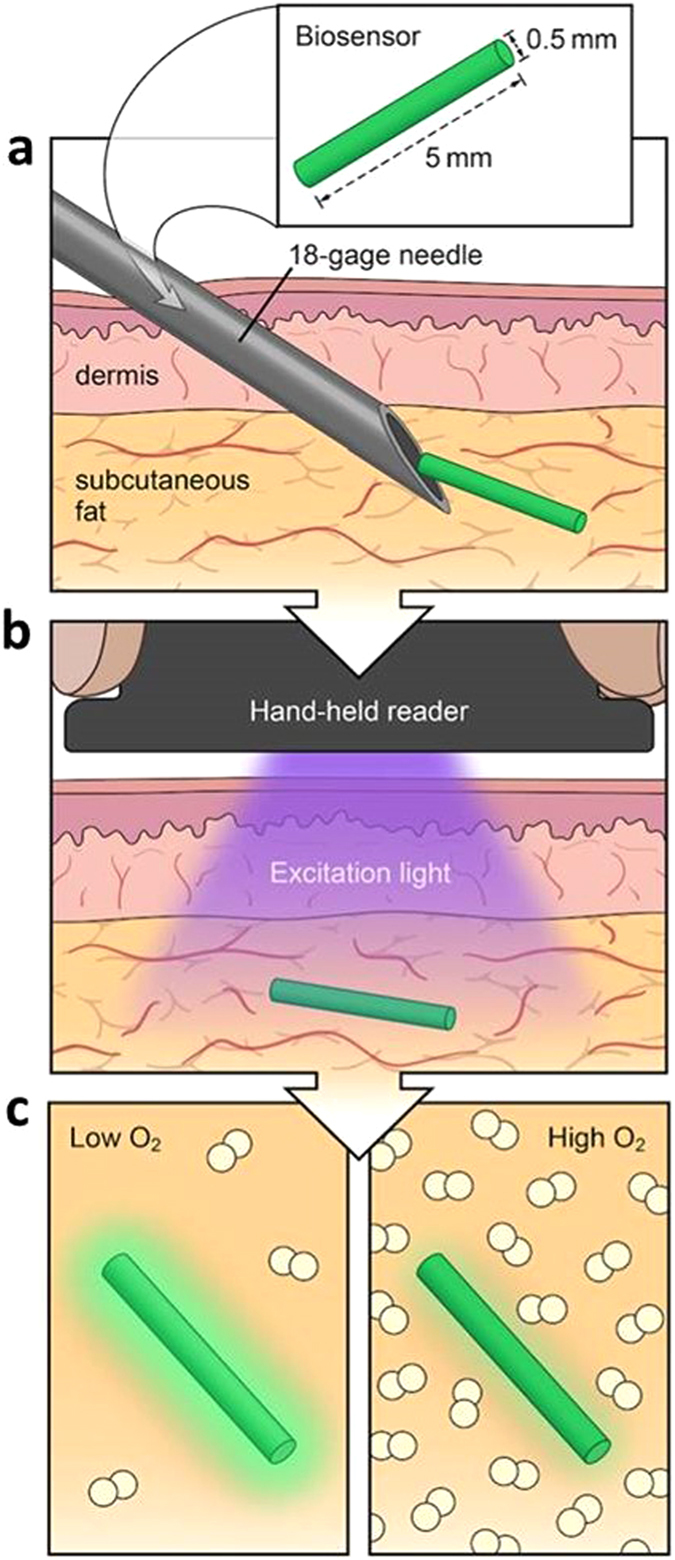
Injectable phosphorescence biosensor overview. (**a**) Small size (5 mm × 0.5 mm) allows local tissue measurements and injection by an 18 gauge needle. (**b**) Excitation light from reader reaches biosensor. (**c**) Sensor phosphorescence changes depending on oxygen concentration. Illustrated by Lauren Halligan, MSMI; copyright Duke University; with permission under a CC-BY 4.0 license.

In addition to NIR spectroscopy, we employed an injectable phosphorescence-based hydrogel biosensor to measure interstitial tissue oxygenation. Figure 1 depicts the principle of operation of the oxygen monitoring system. The hydrogel sensor detects oxygen based on the time course of phosphorescence quenching by oxygen of metalloporphyrins, a well-established technique with excellent sensitivity and specificity to physiologic oxygen. The phosphorescence lifetime on cessation of the exciting light is inversely related to oxygenation. Eastwood described the principle of using these molecules as optical O_2_ sensors, Vanderkooi *et al*. later developed and tested actual sensors, and Rumsey *et al*. continued to further develop this sensor technology^19–21^. After an initial biosensor injection, the continuous measurement of interstitial fluid oxygenation occurs non-invasively with an optical reader positioned over the skin at the location of the biosensor. The potential clinical use of these biosensors has recently been described for studying tissue oxygenation in critical limb ischemia patients^22^. The purpose of this study was to characterize localized rat hind- limb oxygenation following experimentally induced hind limb ischemia using injected biosensors.

## Results

Throughout the duration of this study, all rats were in good health, with measurements and sensors well tolerated by the body. There was no evidence of pain or erythema at the injection sites, extrusion of sensor, or impairment of limb function before and post femoral ligation or tourniquet application. At sacrifice on Day 84, sensors grossly appeared to be well integrated with tissue and blood vessels with no evidence of a significant foreign body reaction, such as a palpable fibrotic encapsulation or granuloma. All sensors on explanation were found at the original site of injection, thus indicating the absence of significant migration over time.

### *In vitro* Calibration

**Figure 2:**
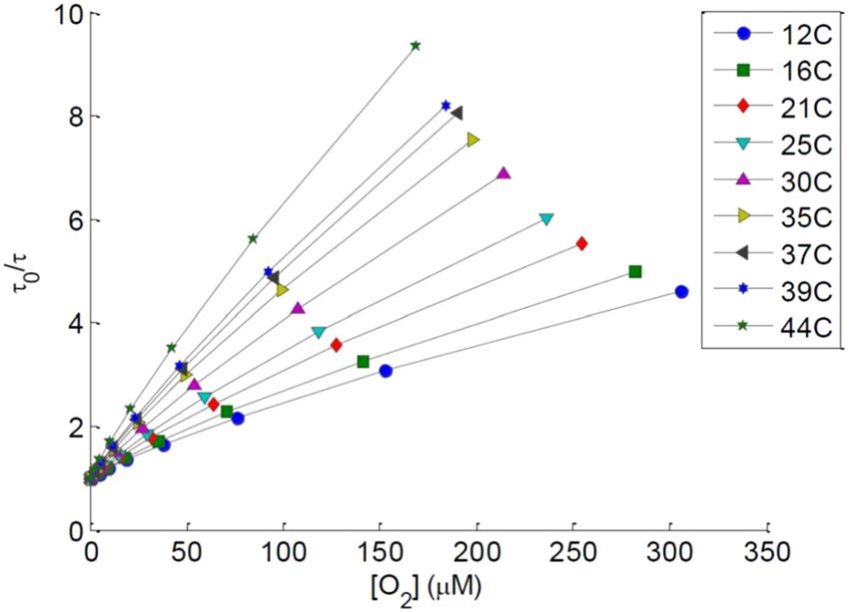
*In vitro* calibration of the biosensors demonstrating phosphorescent lifetime versus oxygen concentration at nine different temperatures.

As the concentration of oxygen in solution measured with Clark electrode increased, the phosphorescent lifetime (τ; see Fig. 2) and phosphorescent intensity were quenched as expected. The phosphorescent lifetime response of the dye exhibited a measurable temperature dependence in the physiological range, also shown in Fig. 2 depicting phosphorescent lifetime versus oxygen concentration and temperature. Similar calibrations were shown by Lo *et al*.^23^. The *in vivo* experiments were conducted in a temperature controlled environment, greatly reducing any likelihood of temperature change causing measurement deviation.

### Phosphorescence Biosensor Integrity

First, the injected sensor integrity was validated by signal reproducibility over time and significant correlation with changes in O_2_ modulation. Paired t-tests between each consecutive O_2_ modulation for phosphorescence biosensors not only show significant differences between high and low O_2_ (p < 0.05) as expected, but also agree with that of the NIR spectroscopy (p < 0.05). Moreover, it was shown that the baseline 100% oxygen values closely approximate those of the recovery 100% oxygen at the end of each experiment, with no significant differences shown (p > 0.4). These findings remained consistent over time, demonstrating stability of the phosphorescence sensors *in vivo*.

### FiO_2_ Modulations

**Figure 3:**
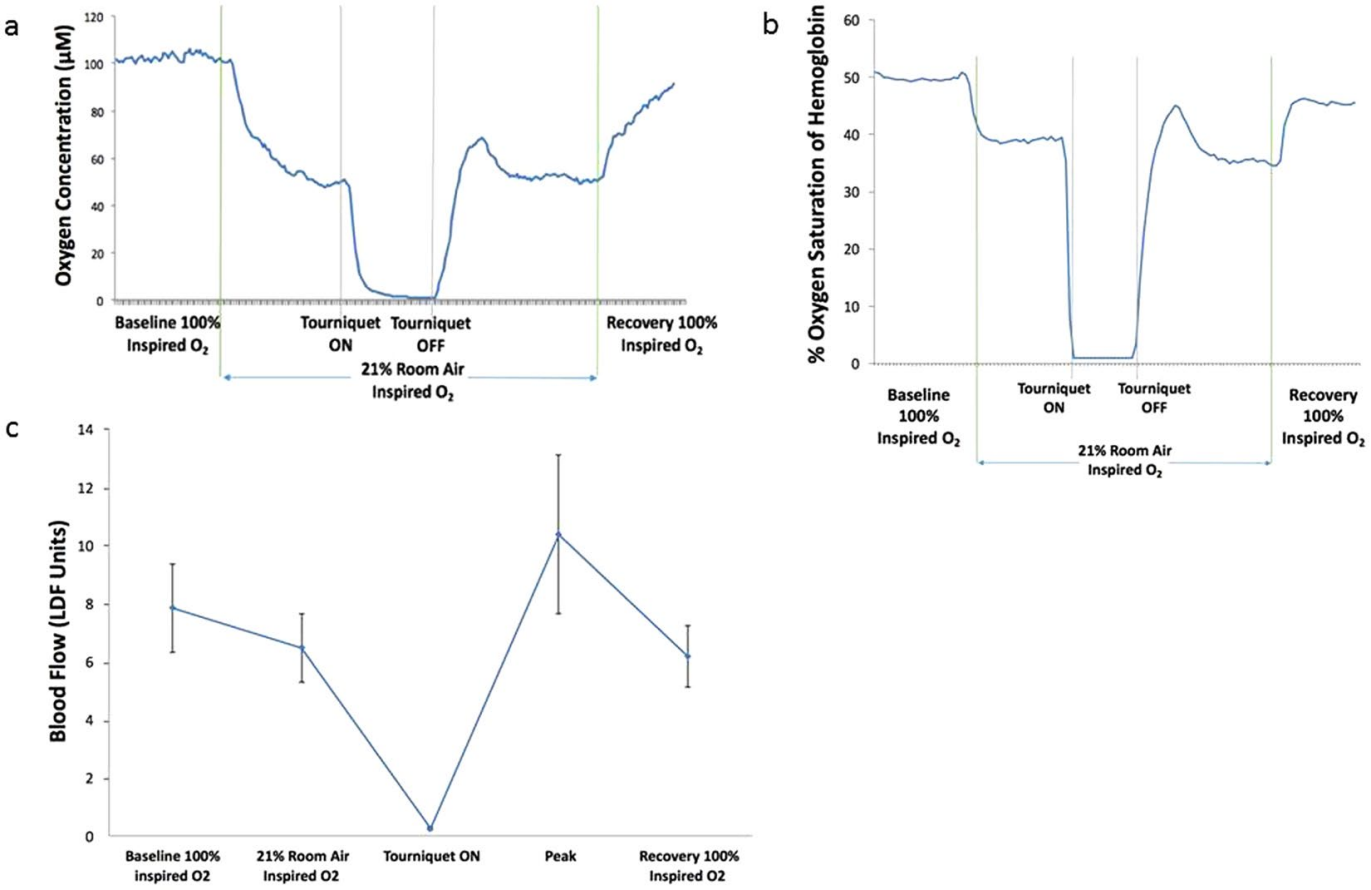
Right hind- limb representative graphs of (**a**) biosensor measurement. (A temporary peak occurs shortly after the tourniquet release), (**b**) NIR spectroscopy (again showing a transient peak after tourniquet release, as seen in the case of biosensor), (**c**) Laser Doppler blood flow (showing mean of three measurements at each stage).

The representative graphs of sequential fraction of inspired oxygen (FiO_2_) modulations for the phosphorescence biosensor, NIR spectroscopy, and laser Doppler flowmetry are shown in Fig. 3a,b and c, respectively. Changing inspired oxygen from 100% to 21% O_2_, all three measurement techniques indicated a significant drop with the exception of the blood flow on D84 in the ligated limbs (p = 0.09). As the inspired oxygen was restored to 100%, the measurements also approached that of the initial baseline on 100% inspired oxygen.

### Tourniquet-Induced Ischemic Challenges

**Figure 4:**
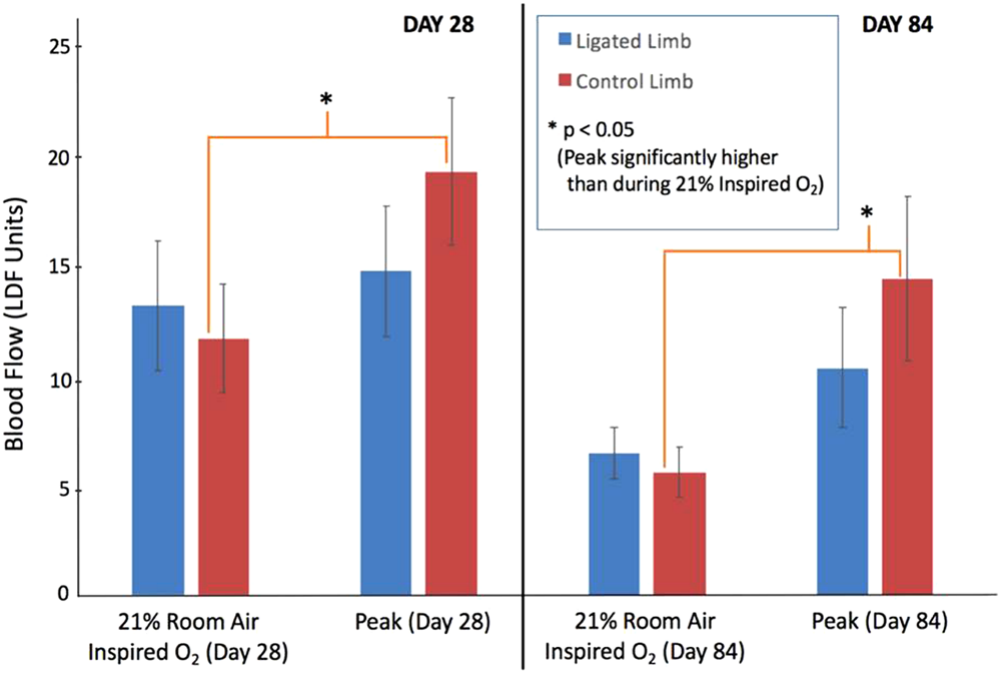
Blood flow during stable period 1 minute prior to tourniquet application (21% inspired O_2_) vs. Reactive Hyperemia Peak. Laser Doppler data for Day 28 (n = 10) and Day 84 (n = 12) both show a peak significantly higher than 21% room air in the control limb, but not in the ligated limb.

All tourniquet challenges were performed while the anesthetized rat was administered 21% O_2_. During simultaneous tourniquet application on both hind-limbs, the respective techniques showed that blood flow and tissue oxygenation (with both biosensors and NIR) all approached zero. Upon release of the tourniquet, we documented with all three measurement techniques that a transient peak above baseline occurred shortly following occlusion release. After peaking, blood flow and oxygenation gradually returned to a steady state similar to the pre-tourniquet 21% inspired oxygen value (Fig. 3). After tourniquet release, the observation of reactive hyperemia was expected based on laser Doppler^24, 25^. Interestingly, whereas the control limb shows a significant difference in blood flow between the peak and pre-occlusion 21% inspired oxygen (*p = 0.04 and 0.01 for Days 28 and 84, respectively), the ligated limb did not (p = 0.66 and 0.24). (Fig. 4).

### Reactive Hyperoxia

**Figure 5:**
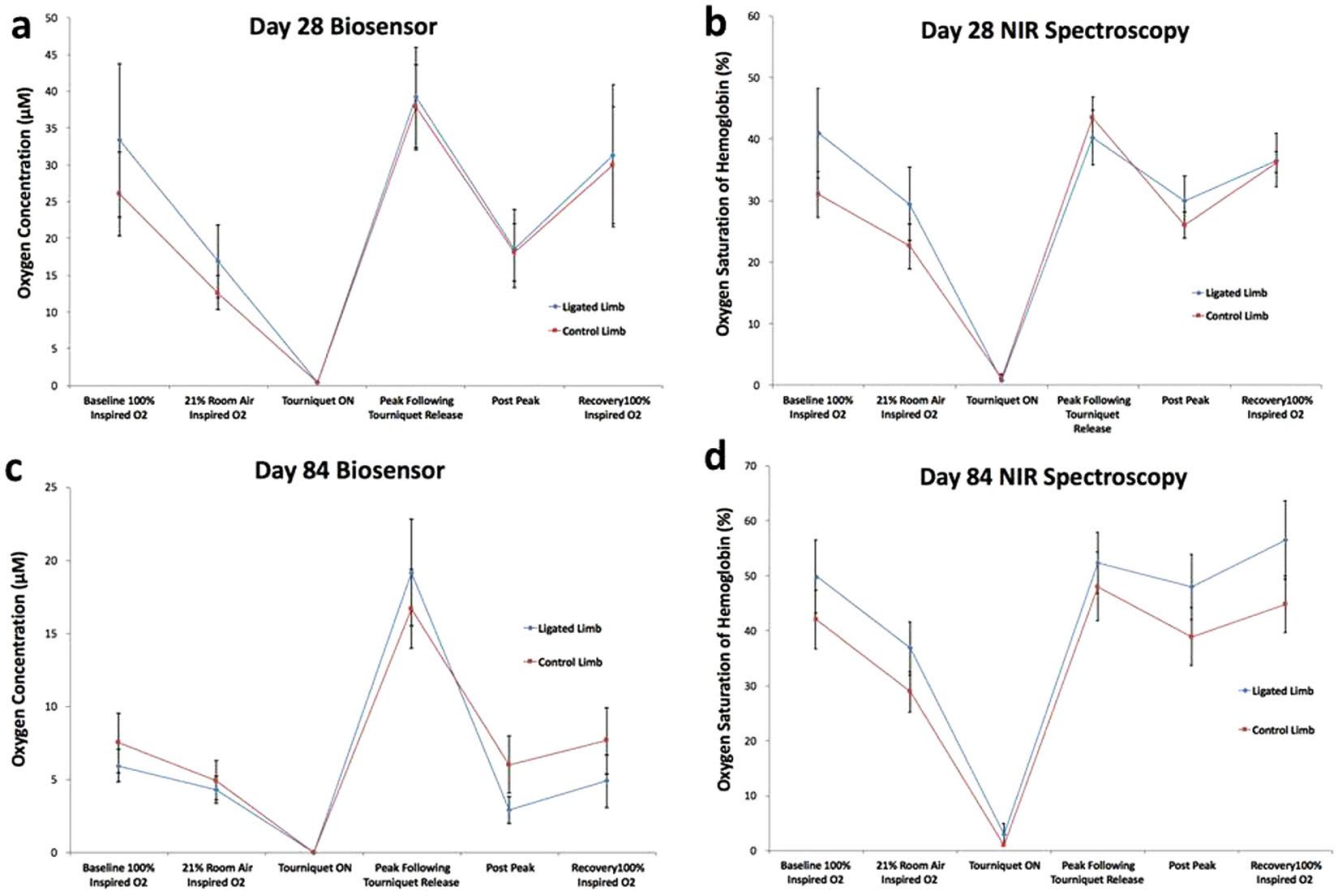
Compiled mean oxygen measurements by day and technique for both limbs: (**a**) Day 28 Biosensor, (**b**) Day 28 NIR Spectroscopy, (**c**) Day 84 Biosensor, (**d**) Day 84 NIR Spectroscopy. Note that techniques demonstrate no significant differences between post peak vs pre-tourniquet and recovery vs baseline on 100% inspired O_2_.

In addition to examining peripheral blood flow, this study further characterized tissue oxygenation post- occlusion. Both the NIR and the phosphorescence data revealed a significantly increased peak value (P) relative to pre- and post-tourniquet steady states. Specifically, we found significant differences in tissue oxygenation between the pre-tourniquet 21% inspired oxygen versus the post-tourniquet peak, as well as between the peak with the post-tourniquet steady state on 21% inspired oxygen (*p < 0.05) (Fig. 5).

### Technique Response Time and Magnitude

**Figure 6:**
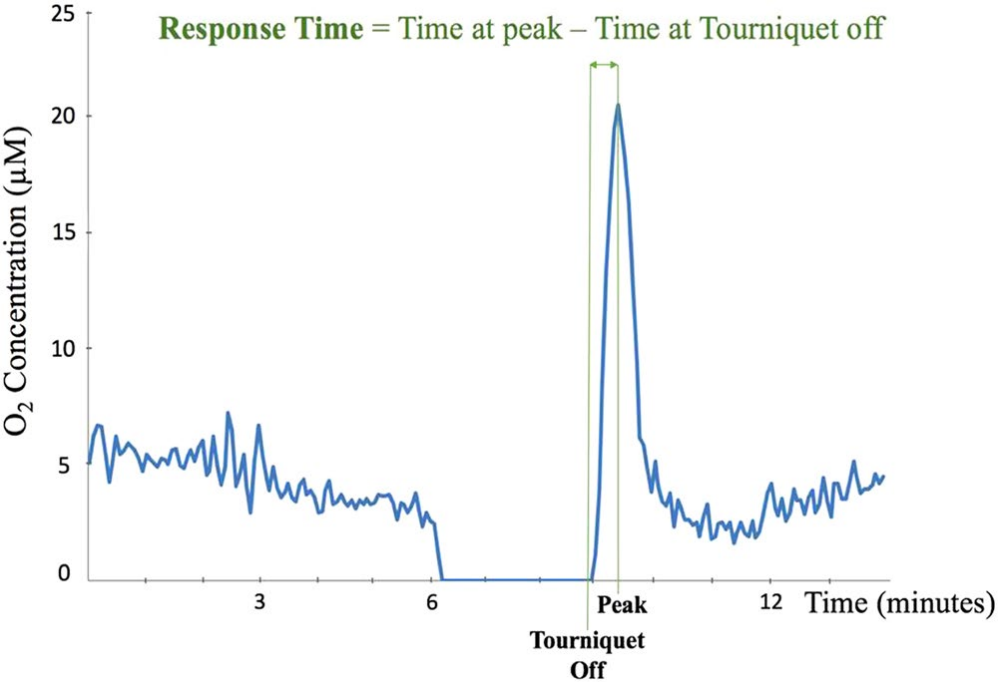
Response time demonstrates the amount of time a technique takes to detect the peak occurrence following tourniquet release.

Results showed no significant difference in response time between NIR spectroscopy and phosphorescence biosensor on Day 28 (p = 0.99; 95.0 secs and 94.7 secs, respectively). (see Table 1 and Fig. 6). But by Day 84, the biosensors achieved the reactive hyperoxia peak significantly faster than the NIR (58.0 sec vs. 177.0 secs for ligated limb, p < 0.05; 80.2 secs vs. 120.0 secs for control limb; p = 0.06). Combining all limbs regardless of whether or not the femoral vessels were ligated, we observed an overall faster response time in phosphorescence biosensors (71.3 sec) than NIR spectroscopy (142.8 sec) on Day 84 (p = 0.0053). However, on Day 28, there was no significant difference in response time between the two techniques (p = 0.99; 95.0 secs and 94.7 secs, respectively). (Table 1, Fig. 6).

**Table 1:** Time to reach reactive hyperoxia peak following tourniquet release.

	DAY 28	DAY 84
Biosensor	95.0 ± 14.2 seconds	71.3 ± 10.3 seconds
NIR Spectroscopy	94.7 ± 14.1 seconds	142.8 ± 19.5 seconds
p-value	0.99	0.0053

Response time to reach reactive hyperoxia peak after tourniquet release. Day 28 shows no difference in response time between the two techniques. However, by Day 84, biosensor shows a significantly faster response than NIR spectroscopy. (n = 15).

**Figure 7:**
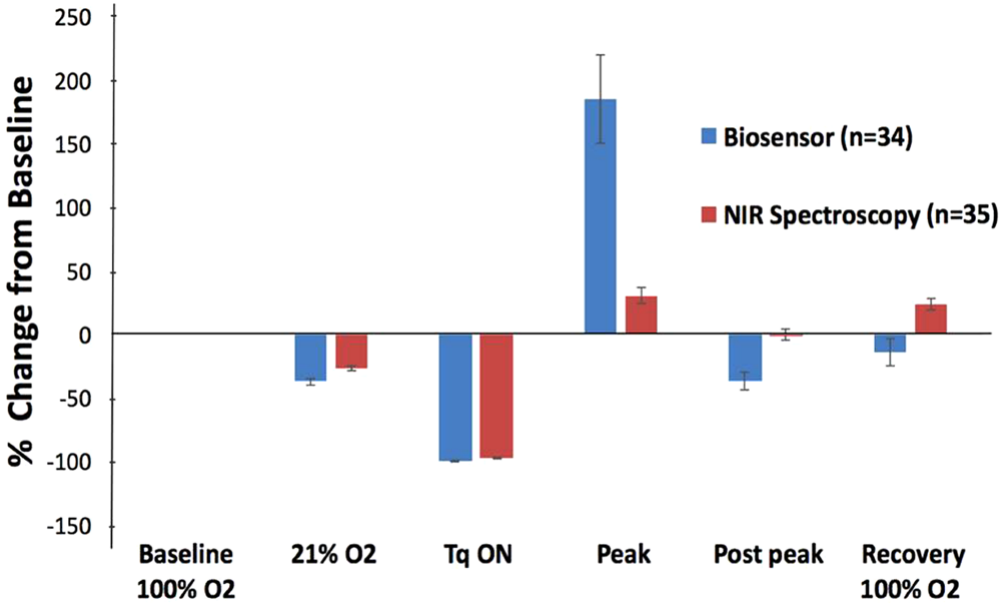
Percent change of each modulation measurement from that of baseline 100% inspired O_2_. Shown are the compiled data for both limbs and both days. The change in magnitude from 21% O_2_ to reactive hyperoxia peak is significantly more pronounced for biosensor as compared to NIR spectroscopy. (p = 0.0065).

In order to compare the magnitude of reactive hyperoxia peaks independent of units of measure, we calculated the percent change from baseline for both the NIR and phosphorescence lifetime measurements. Although both techniques demonstrated the anticipated change with FiO_2_, the phosphorescence biosensor generally exhibited larger relative change. Moreover, the magnitudes of the reactive hyperoxia peaks were more pronounced with the phosphorescence biosensors than the NIR. By combining data for healthy and ligated limbs and time points, we found the overall response magnitude for the peak independent of experimental group was significantly greater with phosphorescence biosensor than with NIR spectroscopy (p = 0.0065) (Fig. 7).

## Discussion

During O_2_ modulations, the significant relative comparisons between values of each consecutive hypoxia challenge were as expected. The decreases from 100% FiO_2_ to 21% FiO_2_ were most prominent in NIR spectroscopy and phosphorescence biosensors, both of which measured tissue oxygenation. Next, the findings of near-zero readings regardless of technique during tourniquet application demonstrated the efficacy of occlusion and therefore validated the significant ischemic insult to the hind-limbs.

For all days and techniques, the absence of significant differences between 100% FiO_2_ baseline and 100% FiO_2_ recovery suggests a consistency of the measurement techniques and a lack of systemic alterations occurring over the course of the experiment. For instance, while dehydration or a decrease in body core temperature could have both prompted decreased peripheral blood flow at recovery versus baseline, that was not observed.

All rats of the study exhibited no gross functional impairment of the ligated hind- limb, suggesting that existing or newly formed collateral pathways met the resting blood flow needs of the hind- limb. Ambulation and eating were not adversely affected, nor was there any gross indication of ischemia. Following occlusion, there was a significant reactive hyperemia in the control limb but no significant hyperemia in the ligated limb, suggesting a limitation or impairment of vasodilatory capacity (Fig. 4). This may suggest that ligation of the femoral artery evoked a dilation of the arterioles to maintain normal perfusion, but also reduced the vasodilatory potential following tourniquet release.

Following an ischemic insult, it is well known that a transient increase of blood flow occurs shortly after occlusion release. This reactive hyperemia might suggest an increase in the supply of oxygen to the affected tissue. However, oxygen consumption and extraction are likely also increased following the occlusion because the “oxygen debt” that is generated during the occlusion needs to be “repaid” post- occlusion. Since the tissue oxygen content is the balance between both oxygen supply and oxygen consumption, it is not obvious if the increases in both supply and consumption would offset, leading to no change in tissue oxygenation. In our study, we observed an elevated oxygenation (hyperoxia), indicating that the increased supply exceeds any increased consumption. To be consistent with the term reactive hyperemia, we have termed this observed transient increase of tissue oxygenation after an ischemic insult as “reactive hyperoxia”.

Many of the ischemic disease etiologies and progressions depend upon the interplay between oxygen consumption and supply within the affected tissues. Monitoring the status of reactive hyperoxia overtime could serve as a more accurate indicator of the microvascular reserve and disease severity. For example, monitoring the reactive hyperoxia in patients with critical limb ischemia may be a diagnostic indicator of tissue ischemia and the responsiveness of microvessels. The ankle-brachial index (ABI), which monitors blood pressure differences between ankle and arm, has been a widely accepted and utilized clinical tool to estimate the blockage of major vessels feeding the distal leg. However, the severity of the peripheral artery disease is often reflected more accurately when under vascular challenge^26^. Under such circumstances, being able to monitor the reactive hyperoxia may better capture the microcirculatory status of such disease. Using transcutaneous PO_2_ measurements, Krajelj *et al*. was not able to demonstrate a significant post-occlusion increased oxygenation, but did observe an elevation using a diffuse NIR spectroscopy technique that probably included substantial muscle in the sample volume^27^. However, the NIR technique could not distinguish which of the tissue beds was being interrogated or dominated the measurement. On the other hand, since oxygen typically diffuses only a few hundred microns from a source (heavily dependent on consumption), the phosphorescence biosensor would be expected to samples a cylindrical volume of tissue within a fraction of a mm from the edge of the injected sensor. That would amount to a volume of approximately 3 µl.

In addition to helping characterize the severity of disease, monitoring of reactive hyperoxia may also assist in investigating the efficacy of treatment for ischemic diseases. One such example could be reperfusion following coronary artery stenting for myocardial infarction. In this case, it might be most desirable to not only restore oxygen supply but also to control the supply to closely match the oxygen consumption profile of the affected tissue and minimize generation of reactive oxygen species and the ensuing reperfusion injury. In other words, the reactive hyperoxia magnitude may quantitatively indicate how much reactive oxygen species is generated.

The observation of tissue oxygenation changes following occlusion could characterize microvascular reserve in a compromised tissue bed. It is becoming more appreciated that quantifying the microvascular response to an ischemic or hypoxic challenge yields a much clearer diagnosis of the health and potential for response of a compromised peripheral bed. Such quantitative assessment may become more commonplace in determining the optimal therapeutic intervention for each patient.

When utilizing clinical tools, it is imperative for the user to understand the basis of what is being measured by the technique employed. NIR spectroscopy is frequently used for monitoring capillary percent oxygen saturation of hemoglobin in surgical flaps^28–32^. The injectable biosensor measures the oxygen concentration in a more spatially localized volume of tissue. Each technique offers various advantages given the appropriate context and specific measurement needs.

The response times of these two techniques were similar at one month. At three months, the phosphorescence technique was able to reflect the peak significantly faster. One possible explanation may be that the microvascular ingrowth into the porous phosphorescence sensor more efficiently delivers oxygen^33^. With regard to response magnitude, the phosphorescence biosensor showed a greater reactive hyperoxia peak. In the clinical setting, such larger magnitude of change could facilitate detection of changes by observers.

Patients with diabetes suffering from peripheral arterial disease and ischemic foot ulcers are often monitored using Doppler ultrasound and ABI. However, this may not accurately reflect tissue oxygenation in the distal target tissue. A toe ulcer may not only have increased oxygen consumption need for healing, but it may have diminished oxygen supply due to diffusion barriers. Traditional non-invasive tests for perfusion (e.g., flow assessment) may be inaccurate due to unique microcirculation changes seen in diabetic foot, along with presence of infection, edema and callus. Even in the presence of palpable pulses and a normal ankle-brachial index or toe-brachial index, the atherosclerosis may still impair the distribution of distal flow to contribute to ulcer development^34, 35^. An injected biosensor may therefore offer a localized and accurate prognosis of the compromised wound bed over time, as well as before and after such interventions as endovascular or bypass surgery.

Another potential area for application of such technique and understanding of reactive hyperoxia is monitoring of tissue survival after ischemia. For instance, transplanted organs and surgical flaps both rely heavily on the degree of oxygenation after restoration of blood supply. Although grossly the tissue of interest may appear well perfused overall, certain locations within the tissue, such the distal tips of flaps and watershed areas, may be compromised due to inadequate oxygenation. The phosphorescence biosensors could serve as a useful predictor of tissue survival. A similar application could be to diagnose tissue where the healing of wounds would be severely compromised and suggest more aggressive therapies.

In addition, the injectable biosensors could also be helpful in providing clinicians and researchers critical information for designing individualized therapies. Due to the rapid proliferation of cancer cells, many therapies take advantage of the high metabolic need of such cells and seek to block the blood supply to the target tumor. Consequently, a biosensor at or near the tumor site may serve to indicate therapeutic efficacy over time and aid in the treatment plan modification. In addition, improving oxygenation of tumors acutely at the time of radiotherapy or chemotherapy often increases the sensitivity of the cancer cells to such treatments.

In summary, this study utilized injectable phosphorescence lifetime-based biosensors to measure interstitial oxygen. The biosensors demonstrated both reactive hyperoxia after tourniquet release and rapid tracking of modulated inspired oxygen. The hydrogel sensor proved capable of localized, real-time, continuous oxygen monitoring over 84 days, and corresponded with measured changes in blood flow using a laser Doppler flowmeter and in local oxygen saturation of hemoglobin using NIR spectroscopy. Consequently, this novel long-term oxygen biosensor may contribute to our understanding, diagnostics, and therapeutic guidance in various hypoxic disorders.

## Methods

### Physiologic Measurements: Blood Flow and Oxygenation

In total, three technologies were used in this study for physiological measurements. Specifically, laser Doppler flowmetry (LaserFlo BPM^2^; Vasamedics, Inc. St. Paul, MN.) was used for detecting blood flow. Near infrared (NIR) spectroscopy (OXY-2; ViOptix, Inc., Fremont, CA) was used to measure tissue hemoglobin saturation. Injectable phosphorescence-based hydrogel biosensors (Profusa, Inc., South San Francisco, CA) were utilized to measure interstitial tissue oxygenation. The hydrogel sensors were prepared using porous poly (2-hydroxyethyl methacrylate) (polyHEMA) as the carrier platform and a Pd-porphyrin as the optical O_2_ sensor that changes both its absolute fluorescence as well as its phosphorescence persistence in an inverse proportion to oxygenation^19, 21, 22^. All measurement devices were secured to the skin using medical tape, and ultrasound gel was applied to optically couple the probe-to the skin for NIR spectroscopy.

### *In vitro* Sensor Oxygen Calibration

Oxygen hydrogel sensors were tested *in vitro* for response to physiologically relevant oxygen concentrations. Sensors were monitored over a range of physiologically relevant temperatures in a closed oxygen controlled system similar in function to that previously described^36^. Rather than using fibers, the excitation source (LED) and detector (silicon photomultiplier) were housed in a cylindrical case (approximately 2.5 cm diameter and 1 cm tall) that was optically isolated except for an excitation and detector port. The case remained in contact with oxygen controlled *in vitro* system for the duration of the calibration. The detector measured both the emitted phosphorescent intensity and phosphorescent lifetime of sensors. Measurements were recorded every 30 seconds as the oxygen concentration was altered between 276 µM and 0 µM by bubbling in varying fractions of air with the balance being nitrogen.

### Animals

**Figure 8:**
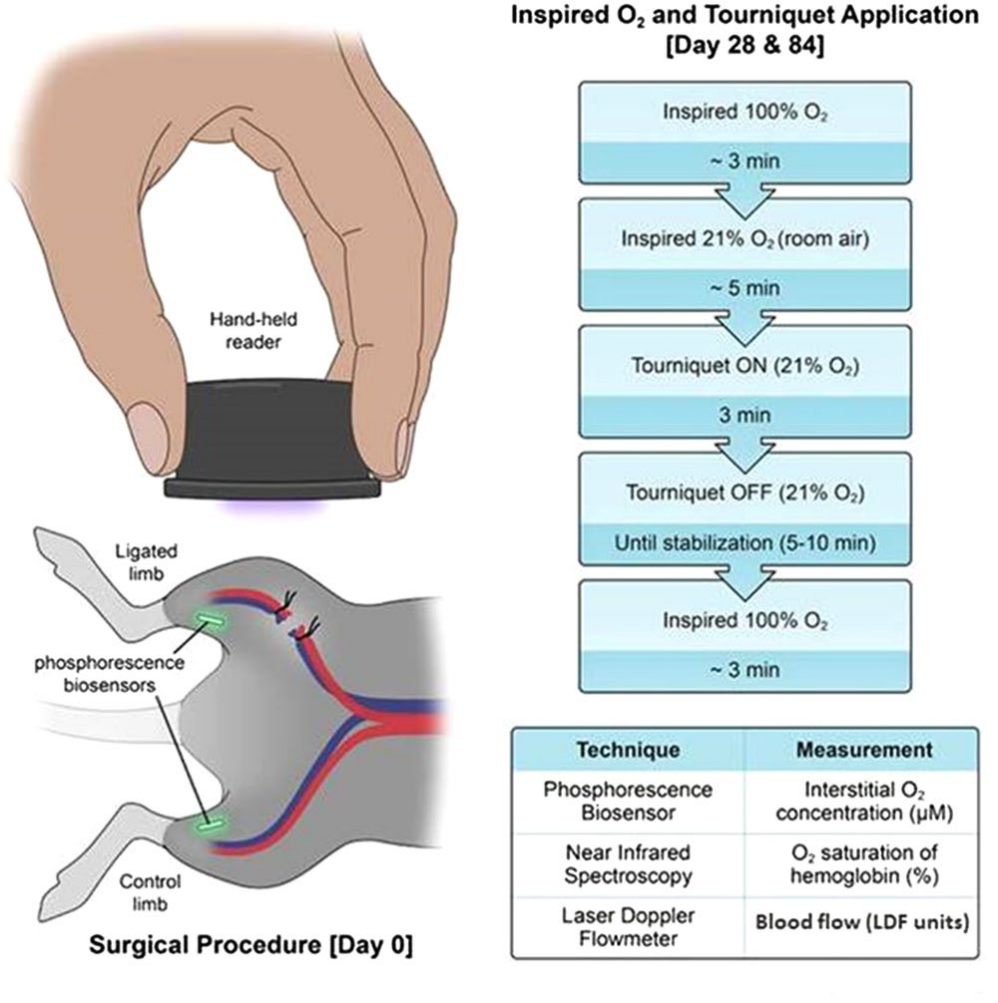
Overview of experimental design. Day 0: bilateral and symmetric subcutaneous implantation of biosensors, with unilateral ligation of femoral vessels on one leg. A similar scheme without the surgery was performed for subsequent measurement days. Days 28 & 84: simultaneous real-time measurement with three techniques (biosensor, NIR spectroscopy, laser Doppler) while the rats were subjected to a series of hypoxic challenges and tourniquet application as illustrated. Illustrated by Lauren Halligan, MSMI; copyright Duke University; with permission under a CC-BY 4.0 license.

All rats were treated according to approved protocols under the Institutional Animal Care and Use Committee (IACUC) of Duke University. All Experiments were performed in accordance with the Guide for the Care and Use of Laboratory Animals of the National Research Council. Male 250–300 gram Sprague-Dawley type rats (Charles River Laboratories, Wilmington, MA) were used. On day 28 and 84, the rats underwent a series of hypoxic challenges through changes in FiO_2_ and tourniquet application (Fig. 8). At the completion of the study, all animals were euthanized with isoflurane anesthesia followed by intravascular KCl injection and bilateral thoracotomy.

### Surgical Procedures

On day 0, each rat was anesthetized under isoflurane (2% adjusted to effect) in 2 l/min oxygen. Bilateral lower extremities and inguinal area were shaved, followed by alcohol and chlorhexidine prepping of the skin in triplicate to create a sterile surgical field. Each rat received bilateral and symmetrical biosensor injection subcutaneously over the gastrocnemius muscle (Fig. 8). Injection was achieved by first retrograde loading each biosensor into the tip of an 18-gauge needle. A sterile metal plunger was advanced anterograde into the needle until it rested gently against the biosensor. Upon positioning the needle over the skin at the desired location, the needle then was advanced at a 30° angle until the tip was located within the subcutaneous plane. The sensor was then deployed by gradually withdrawing the needle barrel while holding the metal plunger in place.

Each rat then received femoral artery and vein ligation on the right side only proximal to the gastrocnemius. To accomplish this, a 1.5 cm incision was created slightly distal to the inguinal ligament in the right inguinal area to expose the femoral bundle (nerve, artery and vein). After carefully separating the nerve from the vessels, 2–0 Sofsilk^®^ ligature (Covidien^TM^-Medtronic, Minneapolis, MN) was used to ligate the vessels at two locations a few mm apart. The vessels were then transected between the ligatures and the skin was closed. The left hind- limb, which did not receive femoral vessel ligation, served as a control.

### Measurements under hypoxic challenge

On post-operative days 28 and 84, the rats were again anesthetized with isoflurane for measurements. For each hind- limb, the laser Doppler flowmeter probe, NIR spectroscopy probe and the optical reader for the phosphorescence biosensor were secured with adhesive tape without compression onto the skin surface in the area of each biosensor to obtain physiological readings.

The rat first breathed 100% inspired oxygen for baseline readings. Once the NIR and phosphorescence biosensor readings stabilized (~3 mins), the inspired oxygen was switched to 21% (room air) as the first systemic challenge. After the readings again reached their new steady state (~5 mins), elastic tourniquets were applied simultaneously to each hind- limb proximal to the ligation site for 3 minutes. The tourniquets were then simultaneously released and the readings monitored until a steady state was again achieved. After the measurements stabilized, the rat was again switched back to 100% to obtain the final recovery readings (~3 mins) (Fig. 8).

### Data analysis

To analyze the laser Doppler flowmeter data, the mean of three measurements, each 20 seconds apart, was calculated within the one-minute period prior to each modulation in FiO_2_ or tourniquet application or release. For NIR spectroscopy and phosphorescence biosensors, the average of the continuous one-minute reading was determined prior to experimental modulation. The post-occlusion peak value (“peak”) was recorded and defined as the maximum value achieved following tourniquet release before the reading began to return to its steady state. Prior to further analysis, data were screened for experimental artifacts, such as an incomplete tourniquet occlusion, an excessive instability of readings, or a complete loss of signal during tourniquet application, which occurred if the optical reader was displaced from the skin. In addition to the magnitude of post-occlusion changes, the time course of the response was also evaluated by recording the elapsed time needed to reach the peak following release of the tourniquet.

### Statistical Analysis

The response magnitude was defined as the difference between the peak value and the steady state value following tourniquet release before switching back to recovery 100% inspired oxygen. Additionally, response time was calculated as the time elapsed from tourniquet release until achieving the peak post-occlusion value. (Fig. 6). Since NIR spectroscopy and phosphorescence biosensors measure tissue oxygenation in different units (% saturation O_2_ vs. μM), these techniques were quantitatively compared by normalizing their respective values into a percentage change between each experimental manipulation and the baseline 100% FiO_2_. Analysis of Variance (ANOVA) and the two-sample paired t-test were performed to determine statistical significance at p-value ≤ 0.05. All values reported within figures and text represent the mean ± standard error of the mean (SEM).
